# Inhibitory Receptors Beyond T Cell Exhaustion

**DOI:** 10.3389/fimmu.2015.00310

**Published:** 2015-06-26

**Authors:** Silvia A. Fuertes Marraco, Natalie J. Neubert, Grégory Verdeil, Daniel E. Speiser

**Affiliations:** ^1^Ludwig Cancer Research Center, University of Lausanne, Lausanne, Switzerland; ^2^Department of Oncology, Lausanne University Hospital (CHUV), Lausanne, Switzerland

**Keywords:** inhibitory receptors, T cell exhaustion, activation, differentiation, immune monitoring, immunotherapy, checkpoint blockade

## Abstract

Inhibitory receptors (iRs) are frequently associated with “T cell exhaustion”. However, the expression of iRs is also dependent on T cell differentiation and activation. Therapeutic blockade of various iRs, also referred to as “checkpoint blockade”, is showing ­unprecedented results in the treatment of cancer patients. Consequently, the clinical potential in this field is broad, calling for increased research efforts and rapid refinements in the understanding of iR function. In this review, we provide an overview on the significance of iR expression for the interpretation of T cell functionality. We summarize how iRs have been strongly associated with “T cell exhaustion” and illustrate the parallel evidence on the importance of T cell differentiation and activation for the expression of iRs. The differentiation subsets of CD8 T cells (naïve, effector, and memory cells) show broad and inherent differences in iR expression, while activation leads to strong upregulation of iRs. Therefore, changes in iR expression during an immune response are often concomitant with T cell differentiation and activation. Sustained expression of iRs in chronic infection and in the tumor microenvironment likely reflects a specialized T cell differentiation. In these situations of prolonged antigen exposure and chronic inflammation, T cells are “downtuned” in order to limit tissue damage. Furthermore, we review the novel “checkpoint blockade” treatments and the potential of iRs as biomarkers. Finally, we provide recommendations for the immune monitoring of patients to interpret iR expression data combined with parameters of activation and differentiation of T cells.

## Definition of Inhibitory Receptors

Immune cell function is tightly controlled and fine-tuned via co-stimulatory and co-inhibitory molecules. Co-stimulatory receptors were originally described to share a common sequence motif called immunoreceptor tyrosine-based activation motif (ITAM) consisting of two YxxL sequences separated by 7–12 amino acids (1, 2). This motif binds Zap70/Syk protein tyrosine kinases via their src homology 2 domain (SH2) (3). A few years later, an inhibitory motif (immunoreceptor tyrosine-based inhibitory motif, ITIM) was discovered in the cytoplasmic tail of Fcγ receptor IIB, a single chain low-affinity receptor for the Fc portion of IgG (4, 5). This receptor could inhibit immune activatory signals by dimerizing with the respective co-activatory receptors on mast cells, T cells, and B cells (6, 7). Like the ITAM motif, the ITIM motif interacts with SH2 domains (8–11). Thereafter, proteins that featured such ITIM motifs and that related to the immune synapse were classified as co-inhibitory molecules or (co-)inhibitory receptors (hereafter referred to as iRs).

The iRs that are nowadays studied in T cells were first described in natural killer cells in the early 90s (12, 13) and were defined as follows (3, 5, 12, 14, 15): (a) they possess an ITIM that, (b) when phosphorylated, (c) can recruit SHP1 and possibly SHP2 (1, 2, 9), which (d) in turn interfere with activating receptors to inhibit downstream activatory pathways (3, 16). At that time, two families of iRs were known: the immunoglobulin superfamily (IgSF) and the c-type lectin superfamily (17–20).

Two of the probably most well-studied T cell-related iRs are programed cell death 1 (PDCD1, also called PD1) and cytotoxic T lymphocyte-associated Antigen 4 (CTLA-4). PD1 was originally discovered by screening for genes that are involved in classical programed cell death in a mouse lymphoid hybridoma and a mouse lymphoid/myeloid progenitor cell line (21). The sequence of its human counterpart was described shortly after (22). CTLA-4 was discovered already in 1987 (23) as an immunoglobulin receptor, but its function was only determined in the mid-90s.

Today, the definition of iRs has changed from the above-mentioned features to a rather functional definition: most surface receptors that are classified as iRs contain an ITIM, such as B and T lymphocyte associated (BTLA) and PD1. But in addition, receptors that lack an ITIM are recognized as iRs, making the first characteristic above (a) no obligation. A prominent example is CTLA-4, which contains a Tyr–Xaa–Xaa–Met motif that is thought to have inhibitory function (12, 24). Also, lymphocyte-activation gene 3 (LAG3) does not feature an ITIM (25). It belongs to the CD4 family and binds to MHC class II with high affinity (26). The most frequent alternative inhibitory motif is the immune receptor tyrosine-based switch motif (ITSM), which also interacts with SH2-domain containing phosphatases such as SHIPs (27–30). ITSMs can be found in the cytoplasmic tail of PD1 and BTLA among other iRs (and in addition to ITIMs). A summary of the inhibitory sequence motifs on iRs including less frequent motifs can be found in the review by Odorizzi and Wherry (31).

The new functional definition describes iRs as co-inhibitory molecules that negatively interfere with T cell activation and function. iRs can inhibit T cell functions at several levels (31): (i) through competition with co-stimulatory receptors for binding to shared ligands or interference in the formation of microclusters and lipid rafts (32–34), (ii) by interfering with downstream signals from co-activatory and T cell receptors (TCRs), and (iii) by upregulating genes that are involved in T cell dysfunction (35). Over the past two decades, the IgSF has grown to include far more than 10 members divided into 8 receptor subfamilies (31, 36). A comprehensive overview of T cell co-stimulatory and co-iRs and their molecular mechanisms can be found in a review by Chen and Flies (36).

## iRs as Hallmarks in “T Cell Exhaustion”

Inhibitory receptors were set at the forefront of research interest with the description of their involvement in the phenomenon of “T cell exhaustion”. The phenomenal clinical impact of the therapeutic blockade of iRs to restore T cell function in cancer (as outlined in the last parts of our review) has greatly contributed to the widespread acknowledgment of the functional importance of iRs. “T cell exhaustion” was coined to describe the functional state of CD8 T cells that persist but show poor effector functions in mice chronically infected with LCMV Clone 13 (37). The molecular signature of exhausted CD8 T cells was later characterized in this prototypic mouse model of LCMV infection, comparing the phenotype and functionality of CD8 T cells in chronic (Clone 13 strain) versus acute (Armstrong strain) infections (38). As opposed to acute infection, where effector CD8 T cells give rise to memory cells once the pathogen is cleared, persistent antigen stimulation and inflammation in the chronic infection setting leads to progressive loss of function in CD8 T cells, termed “T cell exhaustion”. At the center of the molecular signature of exhausted CD8 T cells was the upregulation of iRs, including PD1, CTLA-4, CD160, and LAG3, setting iRs as hallmarks of “T cell exhaustion” (38, 39). In the field of “T cell exhaustion”, scientific advances were strongly promoted by mouse studies using the prototypic LCMV model (acute versus chronic infection). By contrast, the profiling of exhausted CD8 T cells in cancer was first done in humans, demonstrating that Melan-A-specific T cells in melanoma patients share many molecular features with exhausted T cells in chronic infection (40). Over the last decade, several studies have further linked expression of iRs (e.g. PD1, CTLA-4, TIM3, LAG3, CD160, BTLA, and 2B4) to the phenomenon of “T cell exhaustion” both in mouse models and in patients, in chronic infections, including HIV, hepatitis C virus, EBV, malaria, as well as autoimmune disorders such as systemic lupus erythematosus and in several cancers (41–50). In these various pathological scenarios, expression of multiple and different combinations of iRs has been associated with the exhausted phenotype of CD8 T cells, implying that iRs are not only diverse but also co-regulate “CD8 T cell exhaustion” (51, 52). Consequently, iRs have been generally referred to as “exhaustion markers” (53–55). Importantly, this knowledge on “T cell ­exhaustion” and the implication of iRs has had full impact on therapeutic strategies, with the breakthrough of monoclonal antibodies that block iRs (also referred to as “checkpoint blockade”) to restore T cell function and yielding unprecedented clinical improvements on overall survival of cancer patients. This therapeutic breakthrough of monoclonal antibodies against iRs and “checkpoint blockade” is discussed in the final sections of this review.

## Understanding iR Function: Tribulations through the “T Cell Exhaustion” Field (Authors’ Path, Part I)

In the framework of the characterization of CD8 T cells in cancer, we and others discovered that CD8 T cells in metastasis of melanoma patients had increased levels of iRs, as compared to CD8 T cell counterparts in circulation (40, 44, 48). This increase of iRs at the tumor site correlated with the previously observed decreased functional properties of CD8 T cells in metastasis, as opposed to blood-derived CD8 T cells (40, 56). Therefore, these studies demonstrated that “T cell exhaustion” (increased iR levels and diminished T cell function) also occurs in the context of chronic antigen stimulation and inflammation in the tumor microenvironment (TME) (involving self antigens), similarly to chronic viral infections (involving non-self antigens). “T cell exhaustion” at the tumor site constitutes thus the third stumbling block, in addition to the poor naïve repertoire of self antigen-specific CD8 T cells (low affinity and precursor frequency: first stumbling block) and the poor priming capacity against tumors (inefficient tumor antigen presentation and co-stimulation by tumors: second stumbling block) [reviewed in Ref. (57)].

As mentioned above, multiple studies showed that CD8 T cells in chronic antigen stimulation settings (cancer or viral infections) display diminished function associated with increased iR levels. However, this association (high iR = low function) was neither a direct proof that an iR *per se* provoked lower functionality nor did this association provide mechanistic insights into the function of iRs. In fact, there is as yet limited knowledge concerning iR function and the signaling of the various iRs: what are the precise molecular pathways, the signaling cascades and events downstream of the interactions of iRs with their respective iR ligands? Further to structural considerations whereby iRs contain inhibitory motifs (described above), the evidence on signaling mechanisms is summarized in the reviews by Chen and Flies (36), Baitsch et al. (57), and Odorizzi and Wherry (31).

In order to assess directly the impact of iRs on T cell function, we setup an *in vitro* system to study T cells that express iRs and are exposed to TCR activation surrounded by iR ligands. To control the presence and dose of each iR ligand, and to avoid uncontrolled secondary events from the antigen presenting cell (APC), we made use of artificial APCs (aAPC), namely, beads that could be coated with the desired dose and composition of iR ligands (58). These were cell-sized beads (4.5 μm diameter) covered with epoxy groups that covalently attach any protein (or protein mix). We used anti-CD3 antibody (OKT3 clone) to activate T cells together with combinations of recombinant iR ligands, including human PD-L1:Fc (PD1 ligand), HLA-DR (LAG3 ligand), and HVEM:Fc (ligand of BTLA and CD160). We initially found that beads coated with anti-CD3 and any combination of iR ligands barely activated CD8 T cell clones or primary CD8 T cells to produce cytokines in a 4-h assay, as opposed to beads coated with anti-CD3 only, pointing toward strong inhibition by the presence of iR ligands. However, we performed quality controls of the APC beads and discovered that the procedure used to coat the beads (based on standard protocols) lead to the out-competition of anti-CD3 from the surface of the beads upon co-incubation with iR ligands, leading the artifactual “inhibition” of T cell function by iR ligands (in fact, due to less anti-CD3 antibody coated on the beads in presence of iR ligands). After optimization of aAPC bead preparation to obtain beads with equivalent doses of anti-CD3 in absence or presence of iR ligands, the repetition of the experiments revealed that the presence of iR ligands did not result in reduced CD8 T cell function (in clones nor primary cells), neither in 4-h assays of cytokine production nor in proliferation assays for up to 4 days. It is possible that the functional impact of iRs differs depending on the context, for instance, different T cell types may have different susceptibilities to iR-mediated inhibition (“exhausted” CD8 T cells from tumor metastasis may be more susceptible than primary cells from blood of healthy individuals). Several previous studies had investigated iR function using aAPC beads prepared with standard procedures without explicit quality control on the bead coating; our experiments using quality-controlled aAPC beads showed that the mere presence of iR ligands such as PD-L1 did not lead to inhibition of T cell activation (58).

Notwithstanding, in addition to the use of beads coated with T cell-stimulatory antibodies and iR ligands, several other experimental strategies exist to assess iR function. These include the use of T cells over-expressing iRs, stimulated with APC over-expressing respective iR ligands, as well as the T cell functional assays in presence of iR-blocking antibodies.

For instance, the mechanism of PD1 action has been addressed by various experimental means. Wei et al. over-expressed high or intermediate levels of PD1 in primary human T cells by RNA electroporation and stimulated these with aAPC (K562 or T2) over-expressing PD-L1: the different T cell functions tested (cytokine secretion, Ca2+ flux, proliferation) were differentially sensitive to PD1 expression (59). Similarly, using mouse T cells over-expressing PD1 and planar bilayers containing stimulatory molecules in combination with PD-L1, Yokosuka et al. showed that PD1 forms microclusters with the TCR and recruits SHP2 to negatively regulate TCR signaling (60). A multitude of studies have shown that blockade of PD1 and other iRs leads to improved T cell functionality, including cytotoxicity, proliferation, and cytokine production [e.g., Ref. (44, 49)]. Interestingly, in the context of the LCMV infection model of “T cell exhaustion”, PD1-blockade may reverse dysfunction in PD1^int^- but not in PD1^high^-expressing T cells (61), emphasizing again that the levels of iR (high or intermediate) are crucial to determine the impact and strength of its inhibitory action (59). In the case of CTLA-4, several mechanisms of action have been proposed, including inhibitory events downstream CTLA-4 and interference with TCR signaling as well as T cell-extrinsic mechanisms [reviewed in Ref. (62, 63)]. For example, it is known that CTLA-4 is competing with co-stimulatory B7 ligands for binding to CD28. Two models have been suggested, the first in which CTLA-4 increases the threshold for T cell activation and the second in which CTLA-4 attenuates T cell expansion (64). More recently, the efficacy of CTLA-4 blockade in anti-tumor therapy has been at least partly attributed to another T cell-extrinsic mechanism, namely antibody-dependent cell-mediated cytotoxicity (ADCC), whereby regulatory T cells (Tregs) expressing high levels of CTLA-4 are tagged by the CTLA-4-blocking antibody for ADCC by macrophages and depleted within the TME (65, 66). Moreover, an iR might even have opposite functions depending on the molecules that participate to the immune synapse, as is the case for TIM3, which is inhibitory if it interacts with CEACAM-1 and activatory in absence of CEACAM-1 (67). This example shows that it is crucial to consider the remainder of interacting partners, whether co-stimulatory and co-inhibitory, when assessing iR function and CD8 T cell functionality. It also impacts on the design of strategies to block iRs and reverse T cell function, as it might be more relevant to interfere with other interacting partners such as CEACAM-1 rather than blocking TIM3. Altogether, the precise mechanisms whereby each iR works are as yet not fully defined.

Finally, a commonly practiced strategy to address iR function is to assess the functionality of T cells that show positive expression of iRs and compare them to iR-negative counterparts. Several studies have reported that positive expression of iRs (including PD1, CTLA-4, TIM3, LAG3, 2B4, CD160, and BTLA) marked cells that expressed less cytokines (44, 50, 68–72). It is in this comparative exercise that we have found that the consideration of T cell differentiation and activation status is crucial, as will be explained in full detail in the following section.

## iR Expression is Driven by T Cell Activation and Differentiation (Authors’ Path, Part II)

Further to the association of iR expression with “T cell exhaustion” in pathological settings, important insights on the significance of iR expression for the functionality of T cells have been gained from the study of iR expression in CD8 T cells from healthy individuals.

**Figure 1:**
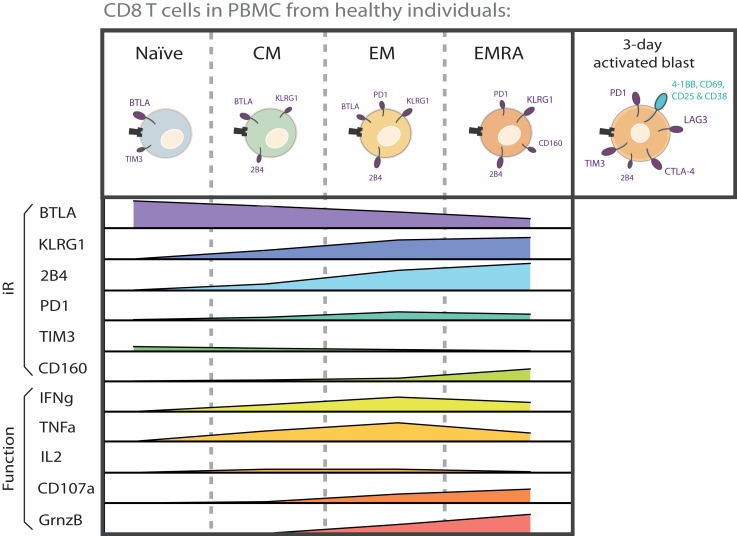
**CD8 T cell subsets are inherently different in iR expression and effector functions**. The various differentiation subsets that are found in circulation in healthy donors are depicted with the iRs that they predominantly express. The relative levels of each iR are indicated below each subset, in parallel to the capacity for cytokine secretion and CD107a translocation. The graphs are based on data from a 6-h intracellular cytokine production assay using PBMC from healthy donors (75). Also shown is iR expression in activated cells (“activated blast”), together with activation markers, based on a 3-day stimulation assay on CD8 T cells from healthy donors (75). The size of the receptor depicted on cells reflects the relative expression of iRs.

Analysis of PBMC from healthy individuals shows, first, that circulating CD8 T cells express various iRs in steady-state, and second, that iR levels clearly vary depending on T cell differentiation (73, 74) (Figure 1). For instance, iRs such as 2B4, KLRG1, and CD160 are expressed at higher levels with increased differentiation. By contrast, BTLA is high on naïve cells and decreases with differentiation. PD1 is particularly expressed in effector memory (EM) cells. TIM3 is present at low levels on naïve T cells. Other iRs such as CTLA-4 and LAG3 are not detectable in steady-state in circulating CD8 T cells from healthy donors.

Importantly, the functional capacities of CD8 T cells also largely vary according to their differentiation stage. For instance, upon stimulation with anti-CD3 and anti-CD28, naïve cells do not produce cytokines, while EM cells are most effective at producing IFNγ and TNFα, central memory (CM) and EM cells at producing IL-2, and EM RA+ (EMRA) cells at expressing Granzyme B and at degranulating (CD107a translocation) [Ref. (75), Figure 1]. The expression of a multitude of other molecules and functions involved in cytokine signaling, cytotoxicity, migration, proliferation, apoptosis, senescence, and stemness are also well known to vary according to T cell differentiation (76–78). There are thus inherent differences in functionality as well as iR expression among CD8 T cell subsets. Therefore, in the study of iR expression and T cell function, it is crucial to keep in consideration that iR expression is tightly linked to the differentiation status, and that this link is already observable in healthy individuals, where it is not related to a particular pathological state nor “T cell exhaustion” (this is further described below in the Section “Physiological Role of iRs to Regulate T Cells in Health and Immune Homeostasis”).

KEY CONCEPT 1. Inherent Differences CD8 T cell subsets are inherently different in the expression of a wide array of molecules and functionalities, including iR expression. iR expression is tightly linked to the differentiation status, and this link is present in healthy immune homeostasis.

In addition to the conventional differentiation subsets Naïve, CM, EM, and EMRA, expression of several iRs has also been assessed in the more recently described Naïve-like stem cell-like memory T cells (SCM) (77, 79). For instance, in the context of Yellow Fever vaccination, we recently showed that human SCM CD8 T cells express high levels of BTLA, very low levels of 2B4, no PD1, and intermediate levels of KLRG1 (79). This iR profile would place them in between Naïve and CM cells in the differentiation gradient shown in Figure 1, as is the case for several other functions that have been addressed by gene expression profiling (77, 79).

**Figure 2:**
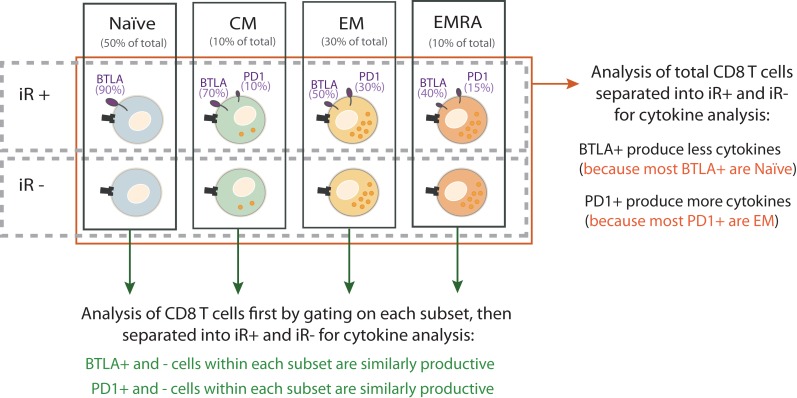
**Discriminating differentiation subsets is the minimal analysis to assess the link between iR expression and functionality of CD8 T cells**. The significance of iR expression on CD8 T cell function can be assessed by analyzing cytokine production by iR-positive versus iR-negative cells. The relative capacity for cytokine production in the various subsets is depicted by the numbers of orange intracellular dots. As an example, the expression of two iRs are shown: BTLA and PD1. The percentage below each subset name indicates the relative frequency of that subset within total CD8 T cells. The percentages next to each receptor indicate the fraction of cells within a given subset that express the receptor (the size of the receptor depicted on cells also reflects relative expression of iRs). These percentages are estimated based on average values in PBMC of healthy donors (75). When iR-positive cells are compared to iR-negative cells within the total CD8 T cells (red arrow), artifactual conclusions can be driven due to the inherent differences in iR expression and effector function of the various subsets. For example, BTLA+ cells in total CD8 T may show less cytokine secretion than their BTLA− counterparts, but this is due to the enrichment of Naïve cells in BTLA+ cells, which do not produce cytokines as compared to the enrichment of differentiated cells in BTLA−. With each subset, BTLA+ and BTLA− are comparable cytokine producers. Therefore, it is not “cytokine production capacity” (=artifactual conclusion) but “differentiation status” what accounts for differences in comparing BTLA+ and BTLA− cells. As a minimal requirement, analysis of individual subsets is necessary to adequately address the link between iR expression and effector function.

The importance of considering T cell differentiation is best depicted by the exercise where we compared cytokine production by iR-positive CD8 T cells versus iR-negative counterparts, either considering the total CD8 T cells (all subsets mixed) or following the separation of subsets (75) (Figure 2). To this end, we used stimulation with anti-CD3 and anti-CD28, importantly in absence of added iR ligands, in order to assess the functionality of iR-positive cells and not the impact of stimulating the iR *per se*. Given that naïve cells do not produce cytokines upon anti-CD3 and anti-CD28 stimulation, the analysis of total CD8 T cells showed highly significant differences between iR- positive versus iR-negative cells in the case of iRs that are inherently expressed at clearly distinct levels between naïve versus differentiated subsets. Yet, these differences were diminished or disappeared when cytokine production was analyzed by comparing iR-positive versus iR-negative cells within each individual differentiation subset (75) (Figure 2). This applies to BTLA (high in naïve) as well as 2B4 and KLRG1 (high in differentiated cells). Interestingly, in general, iR-positive cells were still largely capable to produce cytokines and did not show major impairments as compared to iR-negative counterparts, in agreement with previous reports analyzing PD1 in CD8 T cells from healthy donors (74).

Altogether, this comparative exercise showed that the direct link between positive iR expression and lower cytokine production is generally absent or weak, or only applies to a limited number of iRs in certain CD8 T cell subsets. Moreover, a failure to consider the differentiation status can lead to artifactual conclusions on functional differences between iR-positive and -negative cells.

KEY CONCEPT 2. Artifactual conclusions A failure to consider differentiation status can lead to conclusions that are drawn from wrong argumentation (artifactual) on functional differences between iR-positive and -negative cells. This is illustrated in Figure 2.

We also compared iR expression and function in CD8 T cells from tumor-infiltrated lymph nodes (TILN) in melanoma patients with similar results, in agreement with previous studies where we showed that PD1+ CD8 T cells from PBMC from melanoma patients are not necessarily impaired (80). In the TME (e.g., TILN), however, CD8 T cells can express CTLA-4 and high levels of PD1, and positive expression may correlate with lower cytokine production (69, 75). In our settings, it is technically difficult to compare cytokine levels in iR-positive versus negative cells due to the low cytokine production of CD8 T cells from TILN, although a trend was seen for less cytokines in PD1+, TIM3+, and CTLA-4+ CD8 T cells in TILN (75). Therefore, even in the absence of added inhibitory ligands (only stimulation with anti-CD3 and anti-CD28), the impact of iR expression may be context-dependent, e.g. showing no effect in CD8 T cells in circulation but negative effects in cells from the TME.

In addition, stimulation of healthy donor CD8 T cells for several days with anti-CD3 and anti-CD28 showed that certain iRs are strongly up-regulated, including PD1, CTLA-4, TIM3, and LAG3. Furthermore, such iR upregulation positively correlates with the expression of several activation markers, including 4-1BB, CD69, CD38, and CD25. This indicated that not only differentiation but also the activation status can dictate the levels of iR expression in CD8 T cells.

Of note, the broad inter-individual variability in T cell subset composition, function, and iR expression (75) further aggravates the artifacts in analyzing total CD8 T cells instead of individual subsets separately. This is also aggravated in the case where different samples are compared (e.g., different tissues, infections, time-points) that inherently display different composition of CD8 T cell subsets and where analyses based on comparison of total CD8 T cells undermines variability in differentiation or activation status.

## Significance of iR Expression Beyond “T Cell Exhaustion”

Based on the aforementioned analyses of CD8 T cells in healthy individuals, it became clear that both T cell differentiation and activation were major drivers of iR expression. Consequently, iR expression may, in fact, be an indication of ongoing or recent activation in CD8 T cells as well effective T cell differentiation, in contrast to the association of iR expression with “T cell exhaustion”.

**Figure 3:**
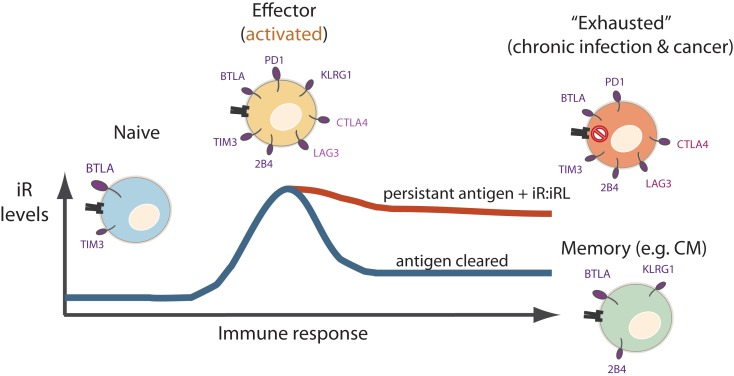
**Levels of iR may peak at the effector phase, and may further modulate differently during acute versus chronic immune responses**. Naïve cells express mainly BTLA and low levels of TIM3. Effector cells express a wider variety of iRs. The levels of certain iRs such as PD1, CTLA-4, LAG3, and TIM3 may peak at the effector phase. Thereafter, iR expression differs in chronically stimulated cells (“exhausted cells”) where iRs are relatively maintained, as opposed to memory cells after clearance of an acute infection where iRs are down-modulated.

Various lines of evidence exist that iRs participate in T cell activation and differentiation, in a “tide model” where iRs can be up-regulated in order to counterbalance co-stimulatory signals following the peak of activation [reviewed in Ref. (36, 81)]. PD1, for instance, was described already in 1996 as a protein that was up-regulated in T and B lymphocytes upon activation *in vitro* (well before it was linked to “T cell exhaustion”) (82). In fact, in the LCMV model of “T cell exhaustion”, iRs are expressed at high levels in the effector phase in both settings, whether in the acute or chronic infection setting (38). Yet, only in the acute infection, the pathogen is cleared leading to contraction of effector cells into memory cells, with the consequent down-modulation of iRs, as opposed to the chronic infection, where the expression of iRs remains elevated. In addition to T cell differentiation, it is important to highlight this “effector/acute” component of elevated iR expression (Figure 3), which has been neglected in the depiction of “T cell exhaustion” models (39) or only recently addressed (36, 41).

In the functional characterization of CD8 T cells, iRs have been used as surrogate markers for T cell dysfunction, in a “guilty by association” reflex where iR expression would mark exhausted cells, even referring to iRs as “exhaustion markers” (53–55). However, multiple studies have directly or indirectly shown that expression of one or the other iR is not always a sign of “T cell exhaustion” but is rather associated with markers or transcription factors involved in T cell differentiation and activation.

KEY CONCEPT 3. Sign of “T cell exhaustion” The phenomenon of “T cell exhaustion” is characterized by T cell dysfunction with elevated levels of iRs. However, iR expression on CD8 T cells is not always a sign of T cell dysfunction and does not necessarily correspond to reduced T cell functions, but may rather mark activated and/or different subsets of functional CD8 T cells.

For instance, high co-expression of PD1, 2B4, CD160, and KLRG1 on HCV-specific CD8 T cells is linked to a CD127 low phenotype (i.e., late differentiation) and recurrent antigen triggering (low epitope diversity) (83). PD1, 2B4, and CD160 are also co-expressed in Flu-, EBV-, and CMV-specific CD8 T cells, where CD160+ but not PD1+ cells are less functional (84). Similarly, in HIV infection, CD160 and PD1 double positive cells are a dysfunctional subset of CD8 T cells, as opposed to cells positive only for PD1 that are rather in an activated state (85). TIM3 expression is linked to an EM phenotype and stronger effector responses in tuberculosis (86). PD1 levels are increased in Acute Friend virus infection yet PD1+ cells are cytotoxic and control infection (87). PD1 correlates with activation markers 41BB (88) in breast cancer and CD38 in HIV (53). In the AT-3 tumor model of breast cancer, PD1^high^ TIM3+ CD8 T cells also express 4-1BB, as well as granzyme B, Ki67, and IFNγ, and persist following radiotherapy (88).

PD1 has also been shown to regulate the development of CM cells following acute vaccinia virus infection in mice: in PD1−/− mice, primary and secondary responses are enhanced and CD8 T cells have a phenotype with high CD62L, CD27, CCR7, and IL-2, which supports that a skewing toward the CM phenotype occurs in absence of PD1 (89). Along the lines of the role of PD1 in differentiation, PD1^high^ CD8 T cells in chronic LCMV infection express high levels of EOMES, in contrast to PD1^int^ that rather express high levels of Tbet (90). Interestingly, the transcription factor FoxO1 sustains high PD1 expression, promoting survival and differentiation of CD8 T cells in chronic LCMV infection (91). The increased levels of PD1, CD160, and 2B4 in total CD8 T cells during chronic HIV infection are also associated with Tbet^dim^ and EOMES^high^ expression (92).

Altogether, in the midst of the implication of iR in “T cell exhaustion”, there is thus also diverse evidence that iRs primarily participate in T cell activation and differentiation.

This has lead to re-evaluations of the significance of iR as “exhaustion markers”, for instance, in the context of SIV infection [where PD1 expression was associated with a CCR7− CCR5+ phenotype rather than dysfunction *per se*, questioning the value of PD1 as a marker of “immune exhaustion” (93)], as well as in cancer [where BTLA and PD1 are proposed to mark cells in a “heightened state of T cell activation” (94)].

## Physiological Role of iRs to Regulate T Cells in Health and Immune Homeostasis

Expression of iRs is physiologically associated with T cell-regulatory events that are not linked to pathological processes. In the context of “healthy” immune homeostasis, iRs co-evolved with co-stimulatory molecules to form a multi-component system of positive and negative regulatory signals surrounding TCR stimulation, which supports the fine-tuning of T cell activation. Possibly, the initial recognition of the target cell via the TCR receptor is priming T cells for response and signaling through co-stimulatory or co-iRs determines the direction and intensity of the response.

KEY CONCEPT 4. Co-evolved with co-stimulatory receptors iRs co-evolved with co-stimulatory receptors to allow for the fine-tuning of T cell responses – as such, iRs are not only present in “T cell exhaustion” but also have a primary role in the regulation of immune homeostasis and preservation of healthy tissue from autoimmunity.

Thus, the expression of co-stimulatory and co-iRs is not mutually exclusive. In the “tide model” suggested by Zhu et al. (81), the rise and fall of waves of co-stimulatory versus co-inhibitory signaling tightly regulates and fine tunes the immune response. The diversity of co-stimulatory receptors and iRs on T cells allows to control the T cell response at every step, from T cell priming, T cell expansion, and contraction, to the various functions of memory T cells. In addition, different co-signaling receptors are important in CD8 versus CD4 T cell subsets.

Pivotal studies in mice have allowed establishing the principles of iR function and their importance for the regulation of the immune response using transgene and (conditional) knock-out technologies, as well as transplantation or cell transfer experiments. Most of the basic knowledge about iRs has been generated and confirmed with such models. For instance, CTLA-4-deficient mice develop lymphoproliferative disease, which leads to tissue damage and ultimately to death within the first month of age (95, 96). In addition to its important constitutive expression and function in Treg cells (97), CTLA-4 is up-regulated following T cell activation (98), and is involved in late stages of T cell priming and systemic activation of T cell responses [reviewed in Ref. (99)].

Like CTLA-4, PD1 expression is induced on T cells by activation (82). PD1-deficient mice develop autoimmune diseases, such as lupus-like diseases or dilated cardiomyopathy (100, 101). When infected with chronic pathogens such as LCMV strain 13 or with *Mycobacterium tuberculosis*, PD1-deficient mice develop strong immunopathological reactions and succumb within days or weeks (100, 102). However, in contrast to CTLA-4 in priming, the PD1-pathway rather plays an important role at the sites of effector T cell activity, by limiting self-tissue damage (101, 103, 104). Upon exposure to target antigen, PD1-deficient T cells show an increased proliferation compared to PD1-expressing T cells (104). This suggests that PD1 is playing a role at later stages of the immune responses, when T cells are already activated.

As described in more detail below, “checkpoint blockade” can provoke immune-related adverse events (IRAE). CTLA-4 blockade for treatment of melanoma patients provoked grade 3 and grade 4 IRAE including autoimmune damages in colon, liver, and hormonal glands (105). The immunological side effects are reminiscent of those in mice that lack Tregs. Indeed, anti-CTLA-4 antibody treatment can lead to Treg depletion via antibody-dependent cellular cytotoxicity (65, 66).

These evidences taken together, it becomes clear that each iR has a specific role at specific events during the tightly controlled immune response of T cells. The plasticity of the immune response requires an interplay of co-stimulatory and co-inhibitory signals in order be executed in a beneficial manner for its host and without causing unwanted damage of healthy tissue. The autoimmune disorders in iR knock-out mice and the IRAE in “checkpoint blockade”-treated patients show that iRs such as PD1 and CTLA-4 are fundamental in the maintenance of a healthy immune homeostasis.

## Breakthrough in Immunotherapy: “Checkpoint Blockade”

During recent years, novel therapies with iR-specific antibodies (referred to as “checkpoint blockade”) have brought major breakthroughs for patients with solid cancers, including melanoma and carcinomas of various organs. This unprecedented success in immunotherapy of cancer is changing the therapeutic principles in clinical oncology, based on the conceptual proof that the immune system of many patients bears the potential to combat malignant disease up to clinically significant levels.

The initial report of clinical benefit for melanoma patients by targeting CTLA-4 (106) was followed by rapid publications confirming and extending these findings through targeting CTLA-4 or PD1 pathways. Two phase 1 clinical trials suggested even further improvements for melanoma patients, by combination therapies with PD1 and CTLA-4 blocking monoclonal antibodies (107, 108). This particular combination causes more frequent autoimmune toxicities than the single agent therapies (109). Nevertheless, the increased clinical benefit for patients with metastatic disease justifies the further development of “checkpoint blockade”. Today, immunotherapy has reached a high level of clinical usefulness. Often, clinical responses in melanoma patients are durable, and some patients are still disease free after many years (110–113). Progress is evident not only in melanoma patients but also in patients with lung and kidney cancer, and more recently bladder and head and neck cancer patients. Currently, there are more than 300 clinical trials registered (www.clinicaltrials.gov) that target iRs. Most of them are using CTLA-4 and PD1/PD-L1 specific antibodies. But other similar approaches are on their way: antibodies against killer cell immunoglobulin-like receptors (KIR) and LAG3 are in early phase clinical studies of novel cancer therapies (NCT01968109, clinicaltrials.gov), and further reagents specific for e.g. TIM3 and BTLA are in preclinical development (114, 115).

## iRs and iR Ligands as Biomarkers

### Biomarkers that may correlate with disease outcome

Major research efforts are made with the aim to identify biomarkers that may help evaluating patient’s prognosis. For many years, tumor immunity parameters were rarely considered as biomarkers for patients with solid tumors. Rather, research focused on other factors related to hormonal or metabolic mechanisms. Even in melanoma, a disease that is since long considered as “immunogenic”, tumor immunity candidate biomarkers played a minor role (116). Now, this has changed, thanks to the new awareness of the importance of immunological mechanisms. Many studies focus on immune cells and their functions. Tumor infiltrating CD8 T cells are of prime interest, because they are frequently associated with better prognosis in the majority of human cancers (117). Currently, there are large multicenter efforts ongoing to determine whether tumor infiltration by activated CD8 T cells can be reliably assessed by standardized methods, and systematically evaluated for eventual routine staging of patients with colorectal cancers (118). The implementation of routine application represents a major challenge. In fact, the vast majority of candidate biomarkers never become routine tools.

During recent years, large efforts have been taken to characterize inhibitory immune receptors and their ligands in cancer patients. Analysis of their expression in the TME revealed highly interesting results [reviewed in Ref. (119, 120)], with much attention having been paid to PD1. In renal cell carcinoma (121, 122), follicular lymphoma (123), and soft tissue sarcomas (124) enhanced expression of PD1 by tumor infiltrating lymphocytes (TILs) was found to be associated with advanced tumor stages and reduced overall survival. Other studies, however, reported that PD1 expression was not associated with clinical outcome [e.g., in melanoma (125)], or correlated with a favorable outcome [e.g., in HPV-associated head and neck cancer patients (126)]. Yet, bad prognostic PD1 expression in one renal cell carcinoma study was linked to Foxp3 and Tregs in TIL (122), showing it is important to concomitantly determine which cell type (and T cell subset) expresses a given iR. Many studies also analyzed the expression of PD1 ligand 1 (PD-L1). Similar to PD1, the results are discrepant. In patients with mismatch-repair-proficient colon cancers, intratumoral expression of PD-L1 was associated with good prognosis (127). A similar finding was made for melanoma (128). In fact, intratumoral PD-L1 expression has been co-localized to infiltrating activated CD8 T cells (129), showing that presence of iR ligands may also reflect ongoing immune responses. However, Massi et al. (130) found that melanoma patients with high-intratumoral levels of PD-L1 expression have a significantly shorter overall survival. Several studies simultaneously evaluated multiple biomarkers. Kim et al. reported that PD1-positive lymphocytes and the expression of PD-L1 predicted poor clinical outcome of patients with soft tissue sarcoma (124). For LAG3, its enhanced expression was found to be associated with poor prognosis of patients with colorectal cancers (131). A similar association was found for patients with chronic lymphocytic leukemia (132). In the case of TIM3, associations with poor prognosis were found for patients with prostate cancer (133), renal cancer (134), gastric cancer (135), and cervical cancer (136). By contrast, TIM3 expression by tumor infiltrating T cells was associated with increased recurrence-free survival in patients with usual vulvar intraepithelial neoplasia (137).

### Biomarkers that may help predicting therapy outcome

Besides the search for associations with prognosis, many of the above-mentioned biomarkers were also evaluated with respect to therapy outcome prediction. The “predictive value” of a given biomarker is high when it correlates strongly with the subsequent response to a specific therapy. This is of particular interest for “personalized medicine”, because better predictions indicate that more efficient and less toxic treatments can be selected for individual patients or groups of highly defined patients. Biomarkers may not only suggest the outcome of a given treatment but can also support the improvement of drug toxicity management.

A major focus is on the role of PD-L1 expression in patients receiving anti-PD1 or anti-PD-L1 antibodies. Several studies reported that clinical responses to PD1-pathway blockade are more likely when tumor cells and/or immune cells express PD-L1 at baseline (138, 139). However, clinical responses to these therapies can also occur in patients where PD-L1 expression is absent or only very low at baseline (140), indicating that they should also have a chance to benefit from PD1-pathway blockade. In fact, currently, there is no biomarker that can be used for patient selection for treatments with antibodies for iRs (140).

Simultaneous assessment of multiple biomarkers may also be useful for predicting therapy outcome. In melanoma samples before treatment with the PD1 specific antibody pembrolizumab, several immune features were associated with subsequent clinical responses, i.e. enrichments of CD8-, PD1-, and PD-L1-expressing cells inside tumors and at invasive margins, with close proximity of PD1/PD-L1, and increased TCR diversity (141).

In humans, the possibilities for assessing functional roles are limited. Nevertheless, cellular assays allow determining functional roles at the level of individual cells, or groups of highly defined cells. Gros et al. found that tumor-reactive T cells *in situ* show increased PD1 expression, more so than expression of TIM3, LAG3, or CD137 (142). Fourcade et al. demonstrated that the combined expression of PD1 and TIM3 identified T cells with diminished functional capabilities (143). However, functional studies in absence of the cells’ natural environment (i.e. outside of the organisms) may give misleading results. As outlined above, T cells that express certain iRs may show reduced function because the receptor indeed mediates inhibition, or because the iR-positive cells are in a different functional state, with different activation and/or differentiation (72).

Triggering of expression of many immune genes is promoted by IFNγ, which is produced by activated immune cells in tumors. PD-L1 is a typical example, and may be taken as a “marker” for immune activation and ongoing CTL activity *in situ* (129). However, PD-L1 can also be constitutively expressed by tumor cells, such as found in a minority of melanoma patients. The combined assessment of PD-L1 expression and CD8 T cells in tumors is currently being used to distinguish scenarios of constitutive versus induced PD-L1 expression, whereby the quantification of CD8 T cells serves as parameter of immune cell activation *in situ* (142, 144).

Overall, it remains difficult to draw conclusions from human studies. iR expression in the TME can be a good sign when ­anti-tumor immune responses are nevertheless taking place and thus contribute to favorable clinical outcomes. As mentioned, iR expression is frequently up-regulated by T cell activation and may be thus a sign of ongoing immune responses. In turn, iR expression may be associated with unfavorable clinical course when occurring in (relative) absence of anti-tumor T cell responses, for example, when tumors constitutively express immune inhibitory molecules (e.g. PD-L1) and fundamentally avoid and or block (almost) any effective immune response against the cancer, even under immunotherapy.

KEY CONCEPT 5. Favorable clinical outcomes The expression of multiple iRs can reflect recent or ongoing T cell activation – iRs may, in fact, mark the cells that responded to a given stimulus (including therapy) and be good prognostic indicators.

## Recommendations for Immune Monitoring of T Cell Function

Biological readouts do often not allow conclusions for individual patients. It remains challenging to identify biomarkers, as most of them end up not being useful for routine clinical management of individual patients. Nevertheless, biomarkers research is often very worthwhile, through their contributions for identifying disease mechanisms.

When analyzing immune responses from patients, caution is required for the interpretation of results. In view of the increasing clinical importance of iRs, patient parameters are now frequently quantified by various methods such as flow cytometry or molecular techniques. From the literature as reviewed here, it becomes clear that the quantification of iR expression by T cells does not allow to conclude that they have impaired functions. Besides considering exhaustion and the TME, it is crucial and possible to analyze parameters of T cell differentiation and activation. Subsets at different stages of differentiation can be distinguished by analyzing T cells with regard to lymph node homing receptors (such as CCR7 and CD62L), and differentiation markers (e.g. KLRG-1 and CD45 isotypes). The separate analysis of naïve cells is most important, but memory cells should also be distinguished from effector cells, as they may also differ largely for iR expression and functions. As mentioned in the previous sections, shifts in iR expression of total cells are often due to changes in differentiation, rather than changes of iR expression within a particular differentiation subset. Similarly, changes in T cell activation are associated with altered iR expression. Therefore, activation markers should also be included, to evaluate this possibility as driver for iR expression.

Regarding the analyses of functional properties, the question remains open whether the available *in vitro* methods allow concluding on *in vivo* T cell functions. The high degree of context dependency and the fact that multiple receptors may simultaneously be involved in controlling T cell function *in vivo* should be taken into account. *In vitro* assays often fail to reproduce the reality. Great care should be taken for drawing conclusions. Nevertheless, in conjunction with results from increasingly sophisticated and realistic mouse models, it is worthwhile to perform functional *in vitro* experiments with human cells, particularly also for the identification of synergistic or antagonistic effects between different iRs and activatory receptors, for example, 4-1BB. It may be useful to characterize the co-expression of multiple iRs, together with other receptors. The recent discovery that TIM3 mediates different functions depending on co-expression of CEACAM-1 (67) represents a typical example for the need to identify possible receptor pairing with functional relevance.

It is necessary to elucidate the contradictory results obtained from studies analyzing iRs and their ligands, and their associations with favorable or unfavorable clinical outcome. Distinguishing tumors in presence versus absence of anti-tumor T cells may lead to a better understanding. Possibly, up-regulation of iRs and iR ligands in the TME in presence of anti-cancer T cells may be favorable indicators because they reflect ongoing anti-cancer immune responses. By contrast, in absence of immune responses, the expression of iRs and iR ligands point to a constitutive immune blockade, which is often associated with unfavorable prognosis and non-responsiveness to immunotherapy.

For the future, advances in basic science are necessary for improved understanding and interpretation of clinical data. For the time being, detailed characterization of T cell activation and differentiation represents already a significant step forward toward a comprehensive characterization and interpretation of iR expression, providing insight in patient’s immune status.

## Concluding Remarks

The success of “checkpoint blockade” therapies in clinical oncology is probably the best proof that iRs can play crucial roles in the regulation of cellular immune responses. However, the mechanisms of action of these novel therapies are still poorly understood, and probably involve multiple cell types and functions. iRs do not only inhibit the effector T cells that express them but can also act early in the generation of an immune response, and/or promote inhibitory functions of immune suppressive cell types such as Treg cells or myeloid cells. Importantly, the sole expression of iRs does not allow to directly conclude on the functional status of T cells. Historically, iR expression was associated with T cell “exhaustion,” i.e. those T cells with reduced effector functions found in chronic infection and in the TME. iRs were considered “guilty by association”, without systematic critical evaluation of their functional impact. Importantly, iRs are also frequently expressed by highly functional T cells such as the ones that predominate in acute infections. This is explained by the fact that T cell activation and T cell differentiation lead to strong upregulation of many iRs, as part of the physiological balance of T cell activation, even though these cells remain highly functional. Clearly, the impact of iRs on T cell function is context-dependent, as the result of the sum of activatory and inhibitory signals. For research and the immune monitoring of patients, it is important to primarily study immune cell functions *in vivo*, and to carefully consider T cell activation and differentiation by subset analyses for the interpretation of iR expression data.

KEY CONCEPT 6. Consider T cell activation and differentiation T cell differentiation and activation are major drivers of iR expression. For research and immune monitoring of patients, it is an absolute need to analyze parameters of differentiation (cell type and subset analysis) and activation markers to interpret iR expression data and functionality of T cells.

## Conflict of Interest Statement

The authors declare that the research was conducted in the absence of any commercial or financial relationships that could be construed as a potential conflict of interest.
